# Pioneering the canine model in microbiota–gut–brain research for social and affective disorders

**DOI:** 10.1002/imo2.70113

**Published:** 2026-06-16

**Authors:** Yun Yu, Qiuhan Wen, Xiaoyang Wang, Mengyu Song, Tifei Yuan, Feilong Deng, Jianmin Chai, Ying Li, Ya‐Ping Zhang

**Affiliations:** ^1^ State Key Laboratory of Genetic Evolution & Animal Models and Yunnan Key Laboratory of Molecular Biology of Domestic Animals, Kunming Institute of Zoology Chinese Academy of Sciences Kunming China; ^2^ Bio‐X Center for Interdisciplinary Innovation Yunnan University Kunming China; ^3^ Shanghai Key Laboratory of Psychotic Disorders, Brain Health Institute, National Center for Mental Disorders, Shanghai Mental Health Center Shanghai Jiao Tong University School of Medicine and School of Psychology Shanghai China; ^4^ Guangdong Provincial Key Laboratory of Animal Molecular Design and Precise Breeding, School of Animal Science and Technology Foshan University Foshan China; ^5^ KIZ‐CUHK Joint Laboratory of Bioresources and Molecular Research in Common Diseases, Kunming Institute of Zoology Chinese Academy of Sciences Kunming China; ^6^ Kunming College of Life Science University of Chinese Academy of Sciences Kunming China

## Abstract

This commentary highlights the translational potential of canine models in investigating the microbiota–gut–brain (MGB) axis, focusing on their relevance to human social cognition and affective disorders, including major depressive disorder and autism spectrum disorder. While traditional rodent models provide foundational knowledge, the unique socio‐behavioral complexity of dogs offers a potential bridge to study the shared neuroendocrine and microbial substrates underlying human psychopathology. This commentary outlines the promising avenues for canine‐based research while critically addressing the practical limitations inherent in generalizing these findings to human populations, including genetic heterogeneity, standardization challenges, and ethical considerations. We propose a phased research pathway incorporating fecal microbiota transplantation and neurobehavioral phenotyping to define a clear trajectory for future translational research. Leveraging this canine platform creates a One Health paradigm, delivering mutual diagnostic and therapeutic advancements for both human and veterinary medicine, with applications for conditions such as anxiety and gastrointestinal disorders.

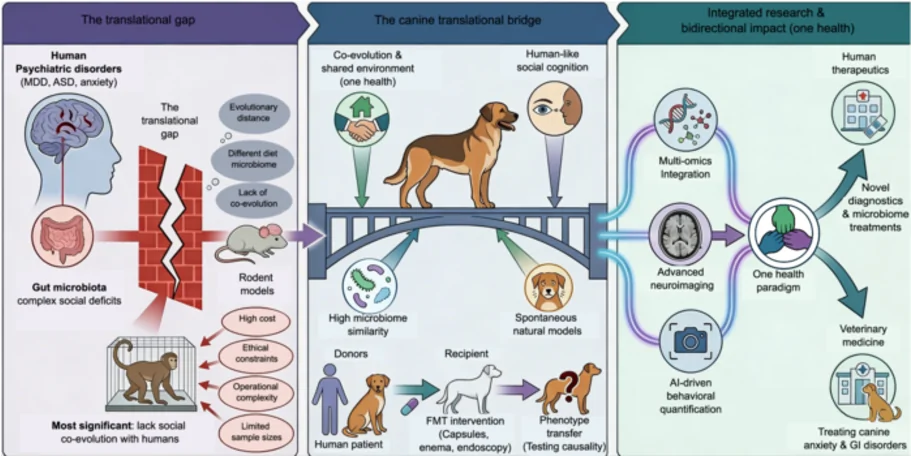

The microbiota–gut–brain (MGB) axis has emerged as a revolutionary paradigm for understanding brain disorders. It is defined as a complex and bidirectional communication network linking the enteric environment and the central nervous system. Over the past decades, research, predominantly in rodent models, has established that gut microbiota can causally influence brain function and behavior, with implications for social and affective disorders ranging from depression and anxiety to autism spectrum disorder (ASD) [1]. These disorders often overlap, as emotional dysregulation frequently leads to social withdrawal or dysfunction. Fecal microbiota transplantation (FMT) from patients with these disorders into germ‐free or antibiotic‐treated mice can recapitulate key behavioral phenotypes. Moreover, symptoms can be rescued or mitigated by FMT from a healthy donor. These demonstrate that the gut microbiome is not merely a correlate but a potential driver of psychopathology [1].

MGB axis is mediated through multiple and synergistic pathways (Figure S1). Yet despite the mechanistic insights (detailed in the supplementary material), clinical translation has been equivocal. Probiotic and prebiotic interventions that robustly modulate behavior in rodents have yielded inconsistent or modest effects in human trials. FMT‐based therapies for psychiatric indications remain largely experimental, with few controlled trials demonstrating clear efficacy [2]. We argue that a critical barrier to translation is the reliance on animal models that diverge substantially from humans in two key dimensions, namely gut microbiome composition and social‐cognitive complexity. Here, we propose the domestic dog (*Canis lupus familiaris*) as a novel translational model that bridges this gap. In this commentary, we argue that the domestic dog represents a strategically underutilized model for MGB translational research, and we outline the evidence, challenges, research priorities, and limitations.

## 1 THE LIMITATIONS OF CURRENT ANIMAL MODELS FOR MGB RESEARCH

Rodents (mice, rats) have been indispensable for foundational MGB research. They have been crucial in identifying fundamental pathways and establishing proof‐of‐concept for microbiota‐behavior causality. However, their limitations increasingly constrain the translation of MGB axis discoveries from preclinical laboratories to psychiatric clinics. Rodents possess simplified brain structures, notably a less developed prefrontal cortex. Their social and emotional behavioral repertoire is limited. Critically, their social sensory world is dominated by olfaction and somatosensation (whiskers), whereas human social cognition is primarily visual and auditory [3]. This fundamental divergence in the primary channels for receiving social information could imply substantial differences in the processing of social signals. Rodent behavioral assays cannot capture the gaze‐based, visually‐mediated social deficits that characterize human psychiatric disorders. Furthermore, rodents differ significantly in gastrointestinal anatomy and gut microbiota composition, driven largely by their disparate diets and body sizes [4]. While humans and mice share the same major phyla (predominantly *Bacteroidetes* and *Firmicutes*), the similarity drops drastically at the genus and species levels. These disparities partly explain why promising preclinical results often fail to translate in human trials [4].

Non‐human primate (NHP) models offer the closest genetic, physiological, and neuroanatomical proximity to humans. However, their widespread adoption for MGB research is severely restricted by prohibitively high costs, stringent ethical constraints, operational complexity (usually under sedation due to low cooperativity), and relatively small sample sizes. Moreover, while genetically close, NHPs do not share a history of co‐evolution with humans. Their intraspecific social cognition differs, and they lack the innate, cross‐species social communicative skills with humans. Their intraspecific cognition is built on competition rather than cooperative communication. In contrast to human social norms, direct and prolonged eye contact in many NHP societies, such as rhesus macaques, functions as a signal of pure aggression or a threat display, often interpreted as a direct challenge to an individual′s social status. While a human might instinctively utilize eye contact to seek reassurance or build rapport, or signal emotional and intentional states, NHPs perceive this same physical gaze through a lens of dominance and confrontation [5]. Such phenotypic divergences fundamentally constrain our ability to model human social deficits, regardless of any conservation in gut anatomy or microbial ecology.

## 2 DOGS COULD OFFER A UNIQUE MODEL FOR MGB RESEARCH

Domestic dog could offer a unique combination of physiological, behavioral, and practical advantages that position it as a potentially ideal, yet underutilized and translational model for MGB research. Dogs have undergone over 30,000 years of unique social co‐evolution with humans, resulting in a specialized social intelligence that is functionally analogous to that of humans in key domains such as communication, cooperation, and attachment (Figure 1A–E). They exhibit extraordinary competence in interpreting human communicative signals, such as pointing gestures and gaze cues, often surpassing NHPs like chimpanzees [6]. This social synergy is best exemplified in the unsolvable task paradigm, where dogs employ referential gazing to actively solicit human assistance, mirroring human social reliance, cooperation, and joint attention (Figure 1A) [7]. Critically, dogs not only recognize human facial expressions, but also exhibit emotional contagion from humans (Figure 1B) [8]. On a neurobiological level, human‐dog eye contact triggers a mutual release of oxytocin, establishing a bidirectional attachment bond that provides a quantifiable readout for social motivation (Figure 1C) [9]. Dogs also display separation‐related anxiety that parallels separation anxiety disorder in children (Figure 1D) [10]. Moreover, dogs display sophisticated language comprehension, including lexical meaning, emotional prosody, and infant‐like “overhearing” mechanisms (Figure 1E) [11]. Recent *SHANK3* knockout Beagle model for ASD exhibits marked social withdrawal, characterized by a persistent avoidance of human eye contact, a diminished interest in social stimuli, impaired joint attention, and attachment deficits, key clinical markers that are hard to be effectively captured by rodent models [12]. These traits demonstrate an extraordinarily high predictive validity in social cognition and behavior using dog models.

**Figure 1:**
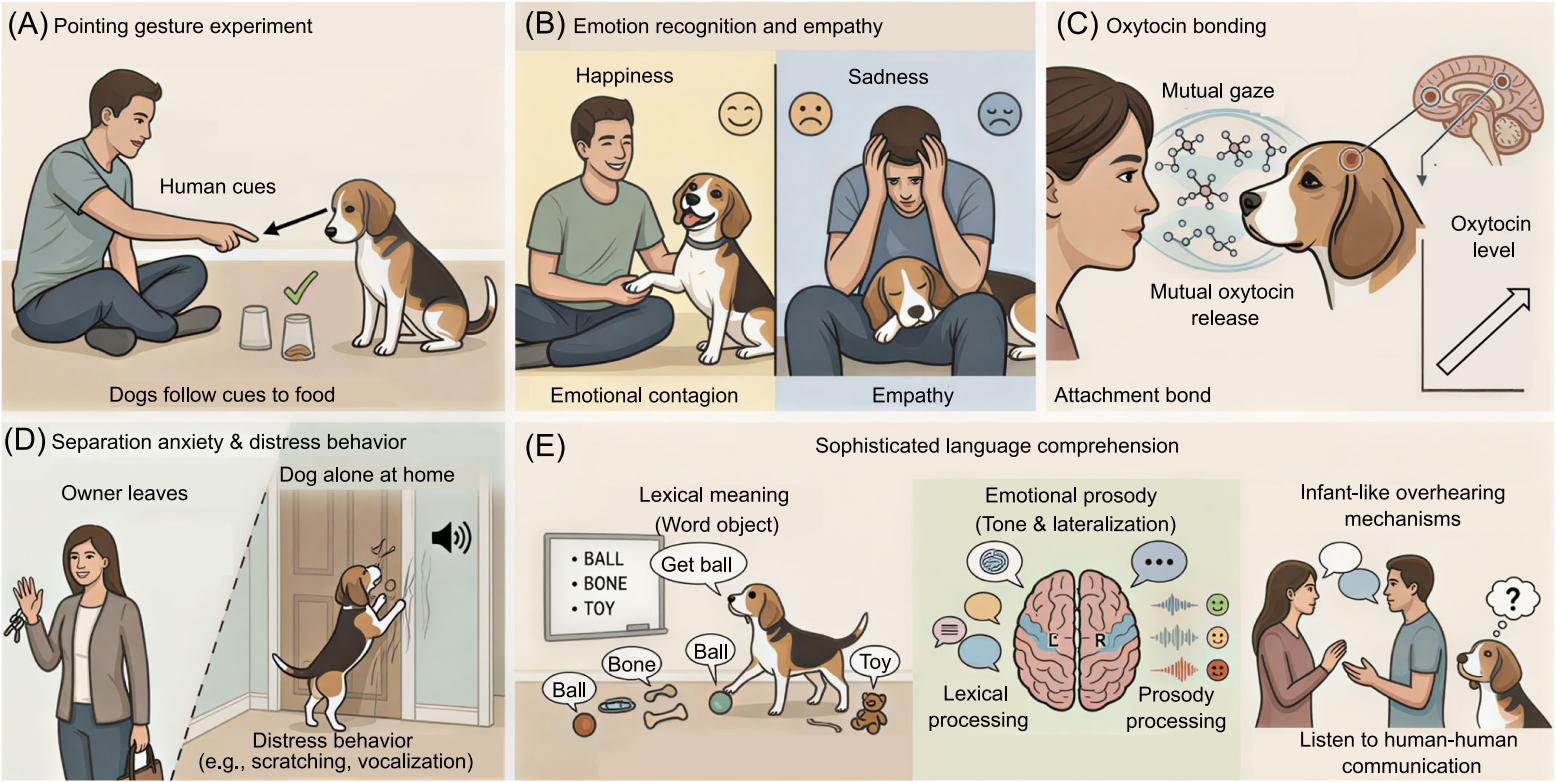
Specialized social intelligence that is functionally analogous to that of humans in key domains such as communication and attachment. (A) Pointing gesture experiment: illustrates a dog's extraordinary ability to interpret human communicative signals. A human provides a pointing cue (pointing gesture), and the dog follows these cues to locate hidden food. This depicts advanced joint attention and functional analogy to human social reliance. (B) Emotion recognition and empathy: depicts the bidirectional nature of human‐dog emotional states. Left: a happy human elicits positive emotional contagion in the dog. Right: a distressed, sad human evokes an empathy response from the dog, which seeks to provide comfort. (C) Oxytocin bonding: illustrates the mutual, positive feedback loop that forms the foundation of the human‐dog attachment bond. Sustained, mutual eye contact triggers the release of oxytocin in both species' brain endocrine systems, quantifiable by increased oxytocin levels. (D) Separation anxiety and distress behavior: depicts the parallels between canine and human attachment disorders. Left: a human owner leaves home, leaving the dog alone. Right: the dog displays distress behavior (scratching at the door, whimpering, vocalization), a conceptual readout for investigating separation anxiety in both children and pets. (E) Sophisticated language comprehension: highlights advanced cognitive parallels, including lexical meaning, emotional prosody, and infant‐like overhearing mechanisms from left to right panel. Lexical meaning: dogs display word‐object association, correctly retrieving an object (e.g., a ball) from a diverse set when verbally commanded. Emotional prosody: depicts human brain lateralization for language processing, where the dog processes lexical content (e.g., word meaning) and emotional prosody (e.g., a person's angry vs. happy tone) using distinct brain pathways. Infant‐like overhearing mechanisms: illustrates a dog's social intelligence by observing and listening to complex human–human interactions, modeling how children learn social language.

Meanwhile, at the level of gut microbiome, a growing body of evidence indicates that the canines exhibit remarkably higher levels of similarities in the composition of genes and related functional characteristics, more so than conventional models such as mice or pigs. Dogs have similar responses in gut microbiota when facing the same dietary change as humans. Wang et al. reported significant convergence in gut microbiota functionality between dogs and humans even compared to NHP macaques [13]. Moreover, laboratory Beagles, specifically, exhibit a gastric pH profile and gastrointestinal transit dynamics that are significantly more aligned with human physiology than those of rodents. This ensures that oral interventions, the most practical clinical route for human patients, can be rigorously validated for survival and colonization efficiency before entering human trials [14].

These dual similarities (social cognition or behavior and gut microbiota composition) may not only enhance face validity by better replicating human symptoms but also position dogs as highly valuable models for capturing the physiological, neurological, and behavioral consequences of MGB interactions, thereby facilitating both mechanistic and translational research.

Currently, canine FMT is an established veterinary clinical procedure (Figure 2A,B). It is employed to restore microbial diversity in dogs with chronic enteropathy [15]. Pilot studies have begun exploring whether behavioral FMT can modulate temperament [16]. Multiple FMT delivery methods are established in veterinary practice, including rectal enemas as the most common clinical approach, oral capsules favored for chronic or behavioral applications due to better compliance, and endoscopic delivery for precise gastrointestinal placement when targeted intervention is required.

**Figure 2:**
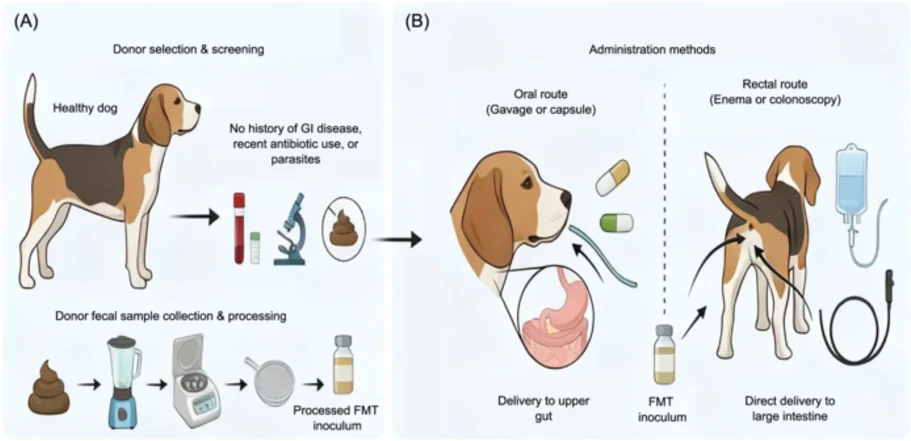
This multi‐panel figure outlines the fecal microbiota transplantation (FMT) process in dogs in veterinary clinics. (A) Donor selection & screening: this panel illustrates the workflow for selecting a suitable stool donor and processing the sample. It includes the screening of a healthy donor dog, stringent health checks, including blood analysis, microscopy, and fecal examination to rule out gastrointestinal (GI) disease, parasites, and recent antibiotic use. Processing flow (bottom) shows the sequential steps from fecal sample collection to blending, centrifugation, and filtration, resulting in the final processed FMT inoculum. (B) Panel administration methods detail the two primary routes for delivering the microbiota to the recipient dog. Oral route (left): shows administration via a gavage tube or oral capsule for delivery directly to the upper gastrointestinal tract (stomach and upper gut). Rectal route (right): illustrates administration via enema solution or during colonoscopy, delivering the processed inoculum directly to the large intestine.

## 3 PROSPECTS AND CHALLENGES

We propose leveraging the FMT technique to construct canine models for social and affective disorders. A critical first step in this endeavor is to develop standardized protocols for depleting the indigenous gut microbiota in laboratory Beagle dogs, which are internationally recognized as a standard large‐animal model in biomedical research. Utilizing broad‐spectrum antibiotic cocktails is a feasible approach. Microbiota depletion in murine models has been shown to elicit profound behavioral shifts, including heightened anxiety and social withdrawal. Yet, these effects remain largely uncharacterized in the canine species. A few pioneer studies have implemented antibiotic cocktails to deplete gut microbiota in dogs. Whittemore et al. (2021) reported that a 3‐week administration of a dual‐antibiotic combination (enrofloxacin and metronidazole) significantly disrupts the canine gut microbiota [17]. However, the study did not evaluate effects on gastrointestinal physiology and behavior phenotypes. More recently, Deschamps et al. used the canine mucosal artificial colon model to simulate the effects of broad‐spectrum antibiotic cocktails on microbial community structure and metabolite production in vitro, which cannot reflect behavioral consequences in live animals [18]. Despite these advances, a critical knowledge gap persists regarding how such microbiota perturbations translate to the systemic physiology and behavioral integrity in laboratory Beagles in vivo.

The next, even more pivotal question to explore is whether gut microbiota derived from humans, or from other dogs with specific behavioral conditions, can effectively induce relevant behavioral changes in canine recipients. By employing FMT to graft microbiota from psychiatric patients, or even high‐anxiety donor dogs, into microbiota‐depleted healthy recipient laboratory Beagles, researchers can test for the inter‐species or intra‐species transfer of core psychiatric phenotypes, such as reduced social initiation, increased avoidance, and impaired responsiveness to human social cues. Conversely, transplanting microbiota from healthy or behaviorally resilient donors into dogs with naturally occurring social or affective disorders could provide a novel therapeutic test of microbial modulation of behavior. Furthermore, it remains an open question whether microbiota from healthy human donors could counteract or rescue behavioral alterations induced by transplantation from human psychiatric patients. These interventional studies, which have yet to be systematically conducted, are critical to validating the dog as a translational model for the gut–brain mechanisms underlying social and affective disorders. Notably, microbiome data analysis in dogs will have to prespecify compositional methods (centered log‐ratio/additive log‐ratio with zero handling), permutational multivariate analysis of variance constrained by batch/breed/household, false discovery rate‐corrected differential abundance, functional pathway inference, and longitudinal mixed models where applicable.

## 4 LIMITATIONS

Several limitations associated with the use of canine models warrant careful consideration. Methodological and statistical challenges stem from the current state of research. For instance, the relatively small number of published canine gut–brain studies restricts the statistical power available for cross‐species comparisons. Furthermore, most existing canine gut–brain studies are observational, limiting the ability to establish causal inference, as interventional studies utilizing rigorous randomized controlled designs remain scarce.

Biological and confounding variables are introduced by the inherent variability in the dog population. Substantial breed diversity introduces confounding factors, including genetic, behavioral, metabolic, and anatomical variability, which complicates cross‐species comparisons and obscures true effect sizes. While the dog shares a human household environment, this shared exposure introduces additional confounders (variable diet, medications, environmental exposures) that are less manageable than in highly controlled laboratory rodent studies.

Model development and measurement hurdles also pose significant challenges. While germ‐free and gnotobiotic dog models are technically feasible, they remain underdeveloped compared to murine models, posing significant cost barriers. Additionally, measuring complex traits like behavior is difficult; while standardized ethological tests and questionnaires exist, their interpretation remains subjective, especially when relying on human judges. Furthermore, observing long‐term effects presents logistical and financial hurdles compared to rodent models, and current limitations in AI‐driven analytical algorithms hinder precise phenotyping in canine studies [19].

Unlike rodents, dogs occupy a special position as companion animals with established human‐animal bonds, raising distinct ethical and animal welfare concerns. Beagles have been the gold standard for laboratory research, especially in toxicology, pharmacology, and neuroscience, for decades. We propose using laboratory Beagle models for MGB research. Using a standardized breed enables comparison between studies and better replication. We further emphasize that all studies must strictly adhere to ARRIVE 2.0 guidelines [20] and employ welfare monitoring protocols that exceed regulatory minimum standards.

However, dogs are not merely research subjects but also patients. Like humans, dogs also spontaneously suffer from various diseases, such as anxiety, that are linked to the MGB axis (described in detail in the supplementary). Consequently, microbiome‐based interventions, such as optimized FMT protocols, can be directly evaluated in pet dogs diagnosed with anxiety, compulsive disorders, or other behavioral conditions. This approach creates a dual‐benefit pipeline in which it offers a direct pathway to novel veterinary therapeutics while simultaneously serving as a robust large‐animal preclinical trial for human psychiatric applications. This reciprocity embodies the One Health philosophy, in which breakthroughs in treating canine behavioral disorders via the MGB axis can directly inform the design of human clinical trials, while insights from human microbiota research can, in turn, refine therapeutic strategies for dogs.

## AUTHOR CONTRIBUTIONS

**Yun Yu**: Conceptualization; formal analysis; investigation; writing—original draft; visualization. **Qiuhan Wen**: Investigation; writing—review and editing. **Xiaoyang Wang**: Conceptualization; writing—review and editing. **Mengyu Song**: Investigation; writing— review and editing. **Tifei Yuan**: Writing—review and editing. Feilong Deng: Writing—review and editing. Jianmin Chai: Writing—review and editing. **Ying Li**: Writing—review and editing. **Ya‐Ping Zhang**: Resource; supervision; funding acquisition; writing—review and editing.

## CONFLICT OF INTEREST STATEMENT

The authors declare no conflicts of interest.

## ETHICS STATEMENT

No animals or humans were involved in this study.

## AI STATEMENT

Nano Banana Pro (Gemini 3.0 Pro Image model) was used solely for generating preliminary conceptual sketches. All content has been verified for accuracy by the authors.

## Supporting information


**Figure S1:** Integrative model of the microbiota‐gut‐brain (MGB) axis: a multimodal communication network.

## Data Availability

Data sharing is not applicable to this article as no datasets were generated or analyzed during the current study. No new data have been generated in this study. Supplementary materials (figures, graphical abstract, slides, videos, Chinese translated version, and updated materials) may be found in the online DOI or iMetaOmics http://www.imeta.science/imetaomics/.
