# Supplementary material for: Pioneering the canine model in microbiota–gut–brain research for social and affective disorders
Source: IMetaOmics. 2026 Jun 16;3(3):e70113. doi: 10.1002/imo2.70113 (PMC13674430; doi:10.1002/imo2.70113)
Supplement: Supplementary file 1 [file IMO2-3-e70113-s001.docx]

Supporting information to

**Pioneering the canine model in microbiota-gut-brain research for social and affective disorders**

**Running title**: From Models to Mechanisms to Therapies

Yun Yu^1#^, Qiuhan Wen^1#^, Xiaoyang Wang^1#^, Mengyu Song^2^, Tifei Yuan^3^, Feilong Deng^4^, Jianmin Chai^4^, Ying Li^4^*, Ya-Ping Zhang^1,2,5,6^*

^1^State Key Laboratory of Genetic Evolution & Animal Models and Yunnan Key Laboratory of Molecular Biology of Domestic Animals, Kunming Institute of Zoology, Chinese Academy of Sciences, Kunming, 650201, China

^2^Bio-X Center for Interdisciplinary Innovation, Yunnan University, Kunming, 650500, China

^3^Shanghai Key Laboratory of Psychotic Disorders, Brain Health Institute, National Center for Mental Disorders, Shanghai Mental Health Center, Shanghai Jiao Tong University School of Medicine and School of Psychology, Shanghai, 200030, China

^4^Guangdong Provincial Key Laboratory of Animal Molecular Design and Precise Breeding, School of Animal Science and Technology, Foshan University, Foshan, 528225, China

^5^KIZ-CUHK Joint Laboratory of Bioresources and Molecular Research in Common Diseases, Kunming Institute of Zoology, Chinese Academy of Sciences, Kunming, 650223, China

^6^Kunming College of Life Science, University of Chinese Academy of Sciences, Kunming, 650204, China

^#^These authors contributed equally: Yun Yu, Qiuhan Wen, Xiaoyang Wang

*Correspondence: yingli@fosu.edu.cn (Ying Li), zhangyp@mail.kiz.ac.cn (Ya-Ping Zhang)

**Figure S1. Integrative model of the microbiota-gut-brain (MGB) axis: a multimodal communication network**.

This schematic illustrates the bidirectional signaling architecture connecting the gastrointestinal tract and the central nervous system (CNS). The vagus nerve functions as a bidirectional superhighway, where sensory afferent fibers transmit real-time data on nutrient sensing and microbial metabolites (e.g., short-chain fatty acids [SCFAs]) to the brainstem, while parasympathetic efferent pathways regulate gut motility and anti-inflammatory responses. The hypothalamic-pituitary-adrenal (HPA) axis orchestrates neuroendocrine feedback, where psychological and physiological stressors trigger systemic responses that alter gut permeability and microbial composition. The immune system acts as a critical mediator; gut microbiota influence barrier integrity and the release of cytokines, which modulate neuroimmune signaling. Central to this network is the metabolic output of trillions of commensal bacteria, which synthesize neuroactive compounds, including SCFAs and neurotransmitter precursors (e.g., for serotonin, dopamine, and gamma-aminobutyric acid [GABA]). These biochemical messengers circulate via the bloodstream or engage neural pathways to fundamentally shape complex host traits, including mood, cognition, behavior, and social drive.

**MICROBIOTA-GUT-BRAIN PATHWAYS**

MGB axis is mediated through multiple and synergistic pathways [1]. The vagus nerve serves as a direct neural superhighway, transmitting sensory information from the gut lumen to the brainstem in real-time. Concurrently, the hypothalamic-pituitary-adrenal (HPA) axis orchestrates neuroendocrine responses to both physiological and psychological stress, influencing gut permeability and microbial composition. Beyond these, the immune system acts as a critical mediator. Gut microbiota modulates gut barrier integrity and immune activity, which in turn affect neuroimmune signaling along the gut-brain axis. Central to this axis is the metabolic output of the gut microbiota. Trillions of commensal bacteria synthesize essential neuroactive compounds, including short-chain fatty acids (SCFAs) like butyrate, and key precursors to neurotransmitters such as serotonin (95% of which is produced in the gut), dopamine, GABA, and norepinephrine. These biochemical messengers, circulating via the bloodstream or neural pathways, convey potent signals to the brain that fundamentally influence behavior, mood, cognition, and social drive [2].

**SPONTANEOUS PSYCHIATRIC CONDITIONS IN DOGS**

Meanwhile, dogs are also patients and suffer from naturally occurring, diagnosable psychiatric conditions with high face validity to human disorders, including separation anxiety disorder [3], various forms of aggression, social anxiety or phobias [4], and canine compulsive disorder (a homolog of human obsessive-compulsive disorder) [5]. Emerging research points to a significant link between the gut microbiota and these behavioral phenotypes in dogs. Gut microbiota composition has been associated with anxiety and aggression in pet dogs [4], and with conspecific aggression in rescued populations [6]. Furthermore, distinct microbial signatures have been identified in dogs displaying generalized fear compared to behaviorally calm individuals [7]. This provides a precious opportunity to study the role of the MGB axis in spontaneous behavior issues and disorders, bridge the gap between induced models and human patients. To realize this potential, however, key mechanistic questions must be addressed. Future research must move beyond correlation to establish causal relationships between specific gut microbes and distinct behavioral outcomes in dogs, and to identify potential bacterial species or functional pathways involved. Moreover, systematic comparative studies with human psychiatric conditions are essential to validate the translational relevance of the canine model and to elucidate conserved mechanisms of the MGB axis across species.

**ADVANCED NEUROSCIENTIFIC TECHNIQUES DEVELOPED IN DOGS**

A comprehensive and well-established methodological framework is now in place for the quantitative assessment of inter-species and intra-species social behavior and the study of its underlying biological mechanisms in dogs. Critically, the exceptional behavioral trainability of dogs enables the deployment of advanced neuroscientific techniques that are often difficult to implement in conventional laboratory animals. These include the use of functional magnetic resonance imaging (fMRI) to map large-scale neural circuit activity [8] and eye-tracking paradigms to investigate attentional and gaze patterns [9]. Together, these well-established methods constitute a robust experimental framework for advancing research in social neuroscience and comparative psychiatry based on dog model.

**REFERENCES**

1. Rusch, Jody A., Brian T. Layden, Lara R. Dugas. 2023. “Signalling cognition: the gut microbiota and hypothalamic-pituitary-adrenal axis.” *Frontiers in Endocrinology* Volume 14: 1130689. <https://doi.org/10.3389/fendo.2023.1130689>

2. Loh, Jian Sheng, Wen Qi Mak, Li Kar Stella Tan, Chu Xin Ng, Hong Hao Chan, Shiau Hueh Yeow, Jhi Biau Foo, Yong Sze Ong, Chee Wun How, Kooi Yeong Khaw. 2024. “Microbiota–gut–brain axis and its therapeutic applications in neurodegenerative diseases.” *Signal Transduction and Targeted Therapy* 9: 37. <https://doi.org/10.1038/s41392-024-01743-1>

3. Yeh, Yu-Min, Xin-Ying Lye, Han-You Lin, Jia-Yi Wong, Chien-Chen Wu, Chin-Lin Huang, Ying-Chieh Tsai, Lih-Chiann Wang. 2022. “Effects of Lactiplantibacillus plantarum PS128 on alleviating canine aggression and separation anxiety.” *Applied Animal Behaviour Science* 247: 105569. <https://doi.org/https://doi.org/10.1016/j.applanim.2022.105569>

4. Pellowe, Sarita D., Allan Zhang, Dawn R. D. Bignell, Lourdes Peña-Castillo, Carolyn J. Walsh. 2025. “Gut microbiota composition is related to anxiety and aggression scores in companion dogs.” *Scientific Reports* 15: 24336. <https://doi.org/10.1038/s41598-025-06178-4>

5. Cao, Xue, Wei-Peng Liu, Lu-Guang Cheng, Hui-Juan Li, Hong Wu, Yan-Hu Liu, Chao Chen, et al. 2021. “Whole genome analyses reveal significant convergence in obsessive-compulsive disorder between humans and dogs.” *Science Bulletin* 66: 187-196. <https://doi.org/https://doi.org/10.1016/j.scib.2020.09.021>

6. Kirchoff, Nicole S., Monique A. R. Udell, Thomas J. Sharpton. 2019. “The gut microbiome correlates with conspecific aggression in a small population of rescued dogs (Canis familiaris).” *PeerJ* 7: e6103. <https://doi.org/10.7717/peerj.6103>

7. Sacchettino, Luigi, Michele Costanzo, Iolanda Veneruso, Valeria D’Argenio, Maria Mayer, Francesco Napolitano, Danila d’Angelo. 2025. “Altered microbiome and metabolome profiling in fearful companion dogs: An exploratory study.” *PLOS ONE* 20: e0315374. <https://doi.org/10.1371/journal.pone.0315374>

8. Prichard, Ashley, Peter F. Cook, Mark Spivak, Raveena Chhibber, Gregory S. Berns. 2018. “Awake fMRI Reveals Brain Regions for Novel Word Detection in Dogs.” *Frontiers in Neuroscience* Volume 12: 737. <https://doi.org/10.3389/fnins.2018.00737>

9. Karl, Sabrina, Magdalena Boch, Zsófia Virányi, Claus Lamm, Ludwig Huber. 2020. “Training pet dogs for eye-tracking and awake fMRI.” *Behavior Research Methods* 52: 838-856. <https://doi.org/10.3758/s13428-019-01281-7>
